# Microbial Consortia Engineering for Cellular Factories: *in vitro* to *in silico* systems

**DOI:** 10.5936/csbj.201210017

**Published:** 2012-12-01

**Authors:** Hans C Bernstein, Ross P Carlson

**Affiliations:** a Department of Chemical and Biological Engineering & Center for Biofilm Engineering, Montana State University, Bozeman, MT 59717, United States

## Abstract

This mini-review discusses the current state of experimental and computational microbial consortia engineering with a focus on cellular factories. A discussion of promising ecological theories central to community resource usage is presented to facilitate interpretation of consortial designs. Recent case studies exemplifying different resource usage motifs and consortial assembly templates are presented. The review also highlights *in silico* approaches to design and to analyze consortia with an emphasis on stoichiometric modeling methods. The discipline of microbial consortia engineering possesses a widely accepted potential to generate highly novel and effective bio-catalysts for applications from biofuels to specialty chemicals to enhanced mineral recovery.

## Introduction

Microbial consortia engineering (MCE) has become an established scientific discipline, populated by biologists, engineers, computer scientists and ecologists [1, 2, 3, 4]. The methodology is based on assembling microbial consortia through enabling, encouraging or enforcing interactions between distinct cell populations and their environment. A common aim of MCE is to capitalize on both the capabilities of individual microbes and their interactions to create useful systems-level emergent properties like enhanced productivity, stability or metabolic functionality [1].

The soundness of the consortia concept for bioprocessing applications is supported by observations in nature. Naturally occurring ecosystems, optimized by eons of evolution, are almost exclusively organized as mixed communities. The consortia-based cell factory concept is in stark contrast with the traditional, albeit successful, bioprocess focus on monocultures and ‘superbugs’ capable of a wide range of concurrent processes. Engineering a single microbe to simultaneously optimize multiple metabolic tasks represents a major challenge under most situations [1]. In fact, the concept of a robust superbug, capable of all functions simultaneously, violates a widely held ecological theory related to stable, competitive ecological function; optimization of one trait typically comes at the price of other traits due to tradeoffs in metabolic resource allocation [5, 6, 7]. Well-designed consortia will almost certainly outperform traditional monocultures. The discipline of MCE possesses a widely accepted potential to generate highly novel and effective bio-catalysts for applications from biofuels to specialty chemicals to enhanced mineral recovery although translation from laboratories to industrial facilities remains a challenge.

## Ecology as the foundation for engineered consortia

Microbial consortia production systems must account for the environmental relationships, distribution and abundance of participating members. Bioengineers are beginning to mine decades of rich ecological theory and experiments to design these templates [5, 8, 9]. Ecological expertise provides a rational framework for dissecting nature's solutions for enabling persistence in diverse environments and for designing theory-based engineered systems. Two established ecological theories are highlighted here because they provide promising design principles for consortial systems.

The first ecological concept is a broad unifying theory based on resource consumption, competition and niche partitioning known as **resource ratio theory** (RRT) [10]; RRT has been described as one of the most successful theories in ecology [11]. This theory is used both qualitatively and quantitatively to assess outcomes between organisms competing for shared, limiting resources. These resource-based interactions can lead to either coexistence or exclusion of competitors. A recent example illustrates how photoautotrophic communities competing for three essential resources (light, nitrogen, phosphorous) can create distinct environmental resource niches which permit coexistence of multiple microbes or the competitive exclusion of all but a single microbe [12]. RRT has been adapted to consider the benefits of resource trading in consortia, highlighting conditions where coexistence is more competitive than monoculture strategies [13]. A major theme from RRT adapted for cooperation is a positive feedback mechanism that creates what has been termed a super-competitor unit; a design goal of many engineered systems [13]. A super-competitor unit is a consortium that possesses the emergent system property of enhanced resource utilization and therefore depletes resources more efficiently than the respective monocultures.

Another ecological theory relevant to MCE is the **maximum power principle** (MPP) initially proposed by Lotka (1922) and invoked in numerous studies including DeLong (2008) [14, 15, 16]. While modifications to the current interpretations of MPP have been proposed [17], the general principle is valuable for analyzing consortial interactions. MMP asserts that biological systems harvest and utilize resources to build and maintain structures and gradients that permit further harvesting of resources. It also dictates that biological systems maximize fitness by maximizing power which is analogous to metabolic rate or the capacity to capture and utilize energy (measured in units of power [J s^-1^]). If a consortium has a higher metabolic rate than the respective monocultures, it will have greater fitness because it possesses a superior ability to acquire available energy. For example based on MMP theory, a consortium that utilizes multiple substrates in parallel would have a higher metabolic rate and therefore fitness than a monoculture that utilized the same substrates sequentially. This common consortia design of parallel substrate utilization is discussed in more detail below. Both RRT and MPP are useful for examining design principles for engineering consortial interactions and provide a solid theoretical framework for testing performance.

## Consortial interaction motifs

Natural consortia interaction strategies typically enable efficient resource usage. At the foundation of many cooperative interactions is **division of labor** through functional differentiation and specialization [18, 19]. Division of labor permits parallel or sequential processing of resources and is often credited with improving accessibility of resources to the community leading to enhanced productivity, nutrient cycling and stability against perturbation. Sequestering different reaction types within designated cells can aid overall resource usage efficiency increasing reaction specificity and reducing the formation of side-products by localizing the reactions to favorable environments. Division of labor also permits concurrent optimization of multiple tasks, a trait useful for multistep-processes like degradation of complex biological material.

A common consortial interaction strategy that occupies a subspace of the classic division of labor motif can be termed **synergistic division of resources** in engineered systems. Chemical species serving as the carbon or energy source (electron donors or electron acceptors) are partitioned between community members in a noncompetitive manner based on metabolic functionality. This template permits parallel processing of substrates and has been used to construct consortia which simultaneously ferment pentose and hexose sugars, a functionality that is often unattainable in monocultures due to catabolite repression [20, 21, 22].

Another common interaction motif is **commensalism**, where one community member's activity provides an ecological niche for others at no benefit or cost to itself. Commensalism is frequent in biofilms where, for instance, the consumption of oxygen by one community member establishes an oxygen gradient creating microenvironments suitable for anaerobic microbes [23, [24, 25]. Another form of commensalism occurs through metabolite exchange when a producer organism secretes byproducts at no benefit or cost to itself which permits sequential consumption by other community members [26].

**Figure 1:**
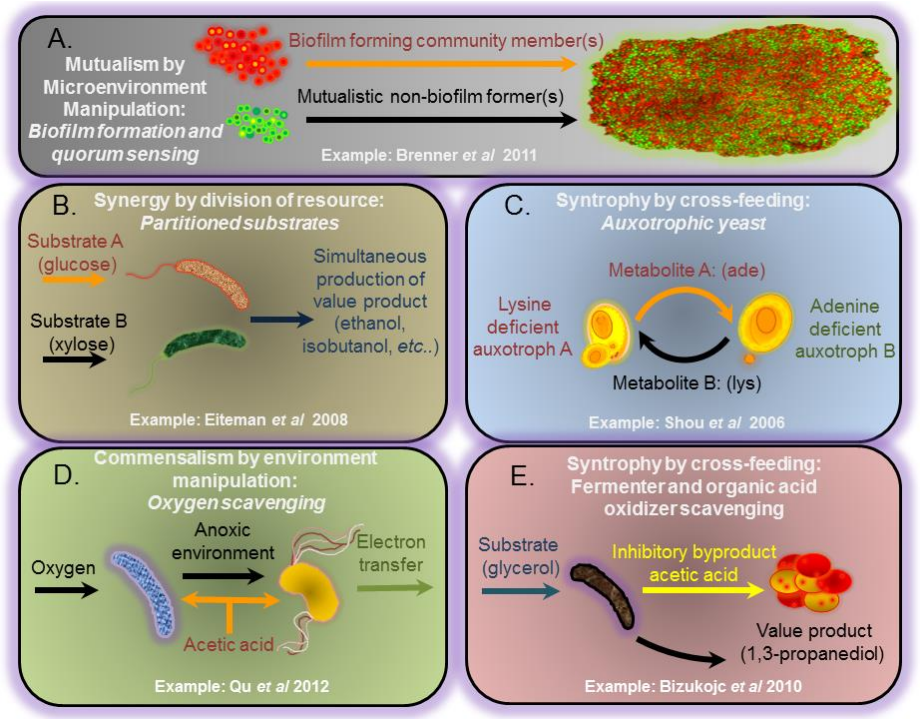
Illustrated examples of microbial consortia organized by common interaction motifs. **A)** A form of mutualism by microenvironment manipulation where one population has the ability to attach to surfaces and create an environment in which a mutaulistic, non-biofilm forming strain can coexist and help support growth of system. For the example presented in Brenner *et al* 2011, this is accomplished via quorum sensing with synthetic cocultures. **B)** An example of consortial co-fermentation of hexose and pentose sugars which highlights synergy by division of resources. **C)** An example of syntrophic cross-feeding in synthetic auxotrophic cocultures. **D)** Oxygen consumption by *Escherichia coli* (blue) aids exoelectrogenic activity of *Geobacter sulfurreducens (orange)* by creating an anoxic environment. This is an example of commensalism by environment manipulation. **E)** An applied example of syntrophy by cross-feeding coupled with organic acid detoxification.

**Mutualistic** motifs are often observed in nature and are defined as relationships that benefit all participants. In cellular factory applications, mutualism can involve **syntrophy**, defined here as resource exchanges or cross-feeding [24, 27, 28, 29, 30, 31]. Mutualistic designs have been utilized in numerous biotechnology studies including consolidated bioprocessing of cellulose coupled with biofuel production [4, 32, 33]. For instance, it is commonly demonstrated in producer-consumer relationships where an organic acid consuming community member scavenges inhibitory byproducts from a producer population [24, 31]. Figure 1 illustrates some common ecological motifs utilized in MCE.

Consortial cellular factory systems are typically designed to express cooperative relationships while excluding competitive, predatory or cheater behaviors. However, interesting synthetic systems have been built to explore these naturally occurring themes [34].

## Consortia types and case studies

**Figure 2:**
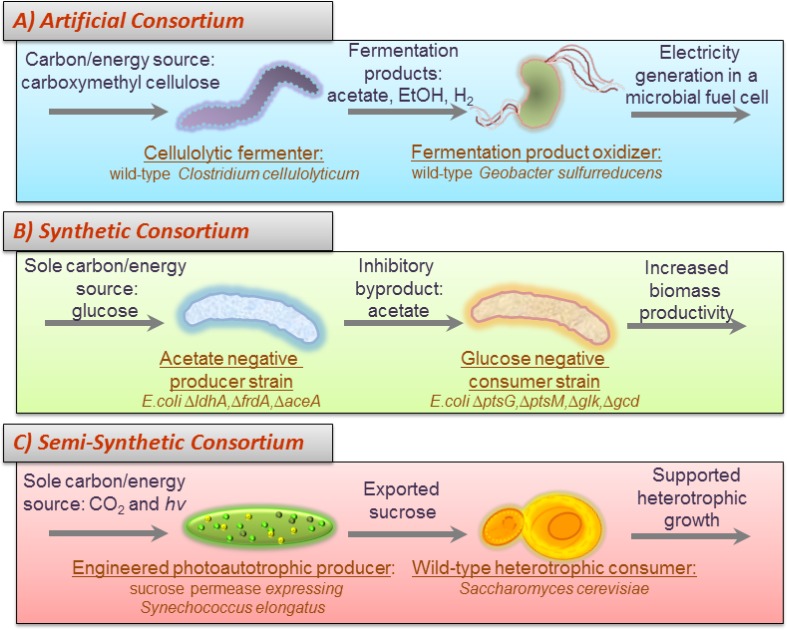
Illustrated examples of engineered consortia categorized as **A)** artificial, **B)** synthetic and **C)** semi-synthetic systems. Artificial communities are composed of wild-type populations which do not coexist naturally. Synthetic microbial communities are composed of two or more metabolically engineered cell populations. Semi-synthetic communities combine metabolically engineered cells with wild-type populations. Illustrations are drawn from cited literature examples; **A)** Ren *et al* 2007, **B)** Bernstein *et al* 2012 and **C)** Ducat *et al* 2012.

The current review discusses a variety of published consortia studies. To organize the current state of the discipline, the published systems are divided into three major classifications: artificial, synthetic, and natural consortia along with one hybrid classification: semi-synthetic consortia (Figure 2). This section highlights recent applications of the resource usage motifs and consortial design strategies through brief descriptions of case studies as well as tables highlighting additional noteworthy studies.

### (i) Artificial Consortia

The term ‘artificial’ microbial consortium (AMC) is used here to describe systems composed of two or more wild-type populations whose interactions do not typically occur naturally. Assembling AMCs require *a priori* knowledge of each population's native eco-physiology; software has been developed to facilitate the pairing of potentially compatible microbes [35, 36]. Industrially relevant AMCs have been applied in multiple areas including renewable energy, food processing and bioremediation [37, 38, 39]. The majority of AMC technologies employ binary cultures while a few studies report purposefully engineered interactions between more than two microbial strains [40].

Industrial interest in alternative energy has driven MCE applications in the areas of biofuels and microbe-mediated electricity generation. Consolidated bioprocessing technologies for conversion of cellulosic feed stocks into biofuels, typically ethanol, commonly employ consortial designs [33]. An AMC example from Xu *et al* (2011) utilized a thermophilic coculture, consisting of *Clostridium thermocellum* and *Clostridium thermolacticum*, to convert either cellulose or glucose/xylose mixtures into ethanol with higher yields than the respective monocultures [41]. The two strains express complimentary cellulose degrading enzymes increasing the accessibility of resources to the binary culture and when fed mixtures of sugars, *C. thermocellum* catabolized glucose while *C. thermolacticum* catabolized pentose increasing consortia metabolic rate relative to the monocultures.

Microbial fuel cells (MFC) are a popular bioenergy platform. A study from Qu *et al* (2012) demonstrated that a coculture of *Escherichia coli* and exoelectrogenic *Geobacter sulfurreducens* could produce more electrical power in an MFC than a monoculture of *G. sulfurreducens* [42]. *E. coli* functioned as an oxygen scavenger and consumed oxygen that leaked into the MFC, a potential problem with anaerobic MFCs [42, 43, 44]. This novel AMC based on commensalism created an environment more conducive for electrical power generation by the oxygen sensitive *G. sulfurreducens*.

Microalgae are becoming popular biofuel hosts because many photosynthetically fix carbon dioxide into energy rich lipids that can serve as biodiesel precursors [45, 46, 47]. Biomass recovery from aqueous media accounts for a large portion of algal-biofuel production costs [48, 49]; an AMC utilized by Zhang and Hu (2012) addresses this challenge using a coculture of microalgae and fungi. *Chlorella vulgaris* was grown photoautotrophically and the filamentous fungi *Aspergillus niger* was added to aid algal biomass collection by causing flocculation [39]. The study does not report a mechanism for syntrophy but photoautotroph-heterotroph pairs are often based on mutually beneficial production and consumption of oxygen and organic acids. Additional examples of AMCs are highlighted in Table 1.

**Table 1:** Specific examples of artificial microbial consortia, respective interaction type and brief description. Examples are ordered based on date of publication.

Consortium Composition and Environmental Context	Interaction Type	Application and Major Conclusions	Reference
Marine fungus, *Pestolotia sp*., cocultured with gram negative bacteria	Competitive interactions	Production of antibiotic, pestalone, by *Pestolotia sp*. in the presence of the bacterial strain CNJ-328	Cueto et al 2001 [78]
Microbial fuel cell cocultures; *Clostridium cellulolyticum* and *Geobacter sulfurreducens*	Commensalism through metabolite exchange	Cellulose degradation by *C. cellulolyticum* produced acetate, ethanol and hydrogen used by exoelectrogenic oxidizer *G. sulfurreducens*	Ren et al 2007 [26]
Papaya juice fermentation with *S. cerevisiae* and *Williopsis saturnus*	Mutualistic division of resource	Fermentation products including complex aroma compounds were produced during coculturing for papaya wine production	Lee et al 2010 [38]
Fermentation of date palm spoilage by *Clostridium acetobutylicum* and *Bacillus subtilis*	Commensalism through micro- environment manipulation	Oxygen removed from culture by *B. subtilis* encouraged fermentation of date palm spoilage by *C. acetobutylicum* to acetone, ethanol and butanol	Abd-Alla and El-Enany 2012 [79]

### (ii) Synthetic and Semi-Synthetic Consortia

A ‘synthetic’ microbial consortium (SMC) is defined here as a system of metabolically engineered microbes which are modified through manipulations of genetic content and/or regulatory processes to establish, encourage or enforce an interaction typically coordinating resource usage. Hybrid systems comprised of wild-type and metabolically engineered populations are defined here as a ‘semi-synthetic’ consortia (semi-SMC). Synthetic and semi-synthetic microbial consortia have been built on many different interaction motifs including metabolite exchange, quorum sensing and synergistic division of resources [20, 24, 27, 30, 50]. Some of the earliest reported SMCs were designed for bioremediation technologies (*see* Table 2) [51, 52, 53]. Several more recent studies describe SMC constructed as artificial ecosystems that have potential to be further developed as bioprocessing platforms [24, 27, 50] while other SMCs have been used directly as catalysts for synthesis of compounds such as lactate or methyl halides [20, 21, 54].

**Table 2:** Specific examples of synthetic and semi-synthetic microbial consortia, respective interaction type and brief description. Examples are ordered based on date of publication.

Consortium Composition and Environmental Context	Interaction Type	Application and Major Conclusions	Reference
Biofilm coculture of engineered *E. coli* and *Pseudomonas putida*	Mutualism through microenvironment manipulation and byproduct scavenging	Multistep detoxification of insecticide by *E. coli* SD2 and *P. putida* KT2440 pSB337 in biofilm	Gilbert et al 2003 [51]
Biofilm coculture of *E. coli* expressing engineered quorum sensing systems	Mutualism though quorum sensing dependency	Developed a quorum sensing circuit-based consensus consortium and engineered co-localization in biofilms	Brenner et al 2007 [50]
Cocultures of auxotrophic *E. coli* deletion mutants	Syntrophy through metabolite exchange	Demonstrated emergent benefits though mutualistic cross feeding of essential metabolites	Wintermute and Silver 2010 [80]
Fluidic micro-droplets containing *E. coli* auxotrophic consortia	Syntrophy through auxotrophic amino acid exchange	Established microfluidic method for rapid screening and compartmentalization of dependent consortia strains	Park et al 2011 [81]

A semi-SMC study by Ducat *et al* (2012) [27] engineered the photoautotrophic cyanobacteria *Synechococcus elongatus* to secrete sucrose which was consumed by a wild-type *Saccharomyces cerevisiae* population (Figure 2). This study reported increased cyanobacteria productivity and carbon fixation rates when sucrose was exported. The effect was attributed to sucrose serving as an electron sink relieving an over reduced cyanobacterial central metabolism. Extracellular sucrose concentrations were reported at >10 mM.

Bernstein *et al* (2012) constructed a SMC system which established a syntrophic producer-consumer relationship between two *E. coli* strains [24, 55]. This study engineered a glucose utilizing producer strain and a glucose negative consumer strain which scavenged metabolic byproducts like acetate [24]. This interaction motif is analogous to strategies commonly found in naturally occurring consortia. Total biomass productivity increased in the SMC compared with monoculture controls even though the SMC ‘metagenome’ was identical to the wild-type monoculture genome. The partitioning and specialization of metabolic function along with a positive feedback mechanism of byproduct detoxification was vital for efficient resource usage. This system also produced spatial partitioning of strains when grown as a biofilm. The glucose negative consumer strain localized primarily to the oxic air interface where it could oxidize non-fermentable byproducts while the glucose positive strain, unconstrained by external electron acceptor availability, was found in micro-oxic and anoxic regions of the biofilm.

The use of SMC to convert renewable resources like cellulosic biomass into value-added bio-products has been the focus of several studies [33]. Bayer *et al* (2009) [54] report a novel semi-SMC technology which produced methyl-halides and synthetic gasoline from cellulosic feed stocks. This study expressed a methyl halide transferase enzyme in a recombinant *S. cerevisiae* strain. The recombinant *S. cerevisiae* was co-cultured with cellulytic *Actinotalea fermentans*. *A. fermentans* catabolized cellulose into inhibitory-acetate and ethanol; the recombinant *S. cerevisiae* converted the acetate into methyl-halides which concurrently detoxified the local culturing environment. Another novel aspect of this study was the coupling of biotic methyl-halide synthesis with abiotic chemical catalysis. *Z*eolite catalysts converted the methyl halides into gasoline-like hydrocarbons. Additional examples of SMC systems are summarized in Table 2.

### (iii) Natural Consortia

The classification of ‘natural’ microbial consortia (NMC) is considered self-explanatory. These systems have extensive industrial applications including bioremediation, wastewater treatment, and biogas synthesis [56, 57]. Highlighted case studies of NMC are not presented here because of an excellent literature base describing their use (*e.g*., Handelsman 1998) [58]. It is worth highlighting a study by Swenson *et al* (2000) that actively guided the development of natural consortia toward a desirable functionality [59, 60]. This approach selects successive generations of laboratory ecosystems possessing improvements in desired functionality. The process, consisting of parental and selected offspring generations, is analogous to monoculture-based adaptive evolution experiments except it is performed at the ecosystem level. The approach has been used to alter ecosystem degradation of industrial chemicals and to enhance plant growth.

## Microbial Consortia in Industry

While the field of MCE has gained popularity in recent years, the use of consortia for industrial purposes is well established. Microbial consortia have been used for commercial production of fermented food products such as vinegar, soy sauce, cheese, and bread for millennia [61]. In addition, consortia-based industrial processes are established in a range of applications including municipal and industrial waste water treatment [62], biogas production [63] and environmental remediation [4]. Consortia are also used in the mining industry to extract minerals from ore [64]. More detailed reviews on existing consortia-based industrial processes can be found in Sabra *et al* (2010) and Bader *et al* (2010) [4, 65].

## *In silico* Analysis of Microbial Consortia

The highly coupled nature of microbial metabolisms and the numerous possible interactions complicates quantitative theoretical examination of microbial communities. Computational analyses are typically required to integrate the large number of metabolic components including hundreds of enzyme catalyzed reactions and interactions into testable formats. Computer models are important design tools and preliminary testing methods for consortial interactions which can save time and money. Traditional microbial ecology modeling approaches have used differential equations, game theory and stochastic methods to gain systems-based insight [66, 67, 68]. Here, the focus is on recent developments in stoichiometric metabolic models which have expanded from their traditional examination of single organisms to microbial communities [9, 69, 70, 71, 72].

Stoichiometric modeling methods are attractive due to their applicability to the growing ‘omics’ databases and because they do not require extensive condition-dependent kinetic parameter sets. These models require only stoichiometric knowledge of system relevant metabolic reactions and the assumption of a pseudo steady-state. The two most widely applied stoichiometric modeling approaches include (i) objective function and constraint-based linear programming (LP) often known as flux balance analysis (FBA) and (ii) unbiased, pathway analysis known as elementary flux mode analysis (EFMA) [73, 74, 75, 76]. Both genome-scale and focused central metabolism stoichiometric models have proven useful as metabolic engineering design tools.

Stolyar *et al* (2007) [71] reported the earliest use of stoichiometric models to study microbe interactions. This FBA study built metabolic models to analyze mutualistic metabolite exchange between a sulfate reducer *Desulfovibrio vulgaris* and methanogen *Methanococcus maripaludis*. This study accurately predicted the relative abundances of *D. vulgaris* and *M. maripaludis* in an experimental coculture.

Another example of FBA being adapted to consortia is found in Hanly *et al* (2011) [70]. The study used a dynamic modeling extension of FBA to simulate two different cocultures engineered for a synergistic division of resources motif to co-ferment xylose and glucose mixtures into ethanol [70]. One study examined a synthetic coculture of *E. coli* mutants while the second system examined a semi-synthetic coculture comprised of a xylose utilizing *E. coli* mutant and a wild-type glucose utilizing *S. cerevisiae*. The *E. coli* coculture simulations were contrasted with experimental data reported by Eiteman et al (2008) [20].

**Figure 3:**
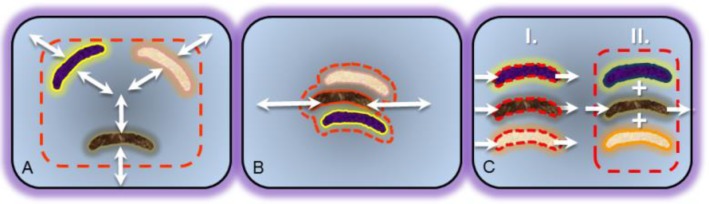
Illustrated diagram representing three computational methods utilized in community elementary flux mode analysis (cEFMA) from Taffs *et al* 2009. The dotted red lines indicate system boundaries for simulations where the interior is constrained by steady-state assumptions and the exteriors account for metabolic sources and sinks. The strategies are categorized as **A)** compartmentalized method in which reactions and metabolites are partitioned into specific species and metabolites can be exchanged through a mass balanced extracellular compartment, **B)** pooled method which combines all ecosystem relevant reactions and metabolites into a single network model without assignment to specific species and **C)** nested method which first computes and identifies ecologically relevant results for individual species-level models and then uses these results to perform a second, community-level simulation.

Community elementary flux mode analysis (cEFMA) has been shown to have its own attributes in the context of metabolic network modeling. Taffs et al (2009) examined mass and energy flows through microbial community models of a well-studied phototrophic, biofilm community [9, 77]. This work developed and compared three distinct methods for evaluating multi-species or multi-functional guild interactions including the use of (i) compartmentalized networks which explicitly accounted for reaction and metabolite partitioning between each specific microbial species, (ii) a collective ecosystem/ metagenomic-level metabolic representation which pooled the metabolic potential of the entire consortia into a single mass-balanced unit with no attempt to assign functionality to individual microbe species, and (iii) a nested, multi-round analysis which first data mined individual microbe-level metabolic models for ecologically relevant strategies, these strategies then served as input reactions for a second round of analysis on a community-level. Each approach had its theoretical and computational advantages and disadvantages but interestingly, a comparison of results across the methods provided additional system insight. For instance, it was possible to quantify efficiency costs associated with the logistics of partitioning ecosystem functionally and then linking the microbes using metabolite exchange. Figure 3 illustrates the three distinct cEFMA modeling approaches developed in this study.

**Table 3:** Specific examples of *in silico* microbial consortia, *in silico* modeling methodology and brief description. Examples are ordered based on date of publication.

Consortium Composition and Environmental Context	*In Silico* Modeling Technique	Application and Conclusions	Reference
Synthetic and semi-synthetic cocultures of *E. coli* deletion mutants grown in batch simulations	Genome scale dynamic-FBA	Systematic evaluation of gene deletions revealed semi-synthetic cocultures optimized for biomass yields and growth rates	Tzamali and Reczko 2008[82]
Mixed microbial cultures from activated sludge in batch reactor simulations	Dynamic-FBA	Comparison of bioplastic production on substrates acetate and propionate	Dias et al 2008 [83]
Syntrophic artificial coculture with *Clostridium butyricum* and *Methanosarcina mazei*	LP/FBA	1,3-propanediol producer *C. butyricum* and syntrophic byproduct scavenging by methanogenic *M. mazei*	Bizukojc et al 2010 [31]
Syntrophic interactions in microbial consortia including a coculture and phototrophic biofilm system described in articles [9, 71]	Multiple Objective-FBA	Established a new FBA framework (OptCom) which permits multiple levels/objectives to investigate consortial interactions	Zomorrodi and Maranas 2012 [84]

## Broader Impact and Future Directions

MCE has become an established academic discipline and the collective capabilities of biologists, engineers, computer scientists and ecologists will continue to push the envelope of this multi-disciplinary field. Additional novel synthetic consortial capabilities will emerge as practical genetic systems become available for unique microorganisms, new ecological theories are tapped, and as consortia construction and control techniques mature permitting systems to be assembled from increasing numbers of interacting components. These advances will obviously be supported by continuing developments in computational systems biology and ‘omics’ based technologies. New MCE technologies will benefit from sustained societal driving forces ranging from fundamental scientific exploration to requirements for new technologies related to sustainable food production, improved resource acquisition like metals from ore, enhanced nutrient cycling of nitrogen, phosphorous and carbon, effective anthropogenic waste management and competitive bioenergy production. Microbial consortia cell factories and MCE have a promising future.
